# Supplementary material for: Interlayer‐Catalyst Method for Metal‐Assisted Chemical Etching of Ultra‐High Aspect Ratio Silicon Nanostructures
Source: Small Methods. 2026 Jul 21;10(16):e70833. doi: 10.1002/smtd.70833 (PMC13505671; doi:10.1002/smtd.70833)
Supplement: Supplementary file 1 [file SMTD-10-e70833-s001.pdf]

Supporting information

Interlayer-catalyst method for metal assisted chemical etching of ultra-high aspect ratio silicon nanostructures

Bryan Peter Jost Benz, Marco Stampanoni, Lucia Romano*

Contents

Figure SI 1: Cross-sectional SEM of commonly used resist showing the reflow problem during Pt deposition

Figure SI 2: Cross-sectional SEM images of a sample prepared with conventional lift-off and one with I-Lo

Figure SI 3: Cross-sectional SEM images of a sample prepared with conventional lift-off.

Figure SI 4: Top-down and cross-sectional SEM of MacEtch experiment with conventional lift-off and Ar milling.

Figure SI 5: Top-down SEM of Pt removed by sonication after a standard lift-off.

Figure SI 6: XPS analysis of an EBL exposed and unexposed samples

Figure SI 7: Cross-sectional SEM of a pinned catalyst

Figure SI 8: Cross-sectional SEM of an Cr/Al_2O_3 interlayer with an undercut created by plasma etching

Figure SI 9: Angle and tortuosity measurements (0.5 cm x 0.4 cm sample in Figure 6)

Figure SI 10: Cross-sectional SEM of the 1 cm x 1 cm patterned sample (second sample in Figure 6)

Figure SI 11: Schematic of the IDONUS tool used

<u>All standard resists show serious issues with reflowing</u>.

Standard lift-off processes were attempted with different options for resists, including the widely used PMMA and MMA as well as CSAR 62. All the patterned profiles deform after the Pt deposition. Despite several attempts to tune the deposition parameters so to record a minimal temperature variation in the chamber, the resist profile is subjected to reflow. The Pt deposition is realized in electron beam assisted evaporator (Bak evaporator from Evatec). It is not possible to cool down the substrate during the deposition, as system is designed to deposit on multiple wafers at the same time. We attribute the deformation to the thermal reflow of the resist during the deposition.

Resist	After development	After Pt deposition
PMMA	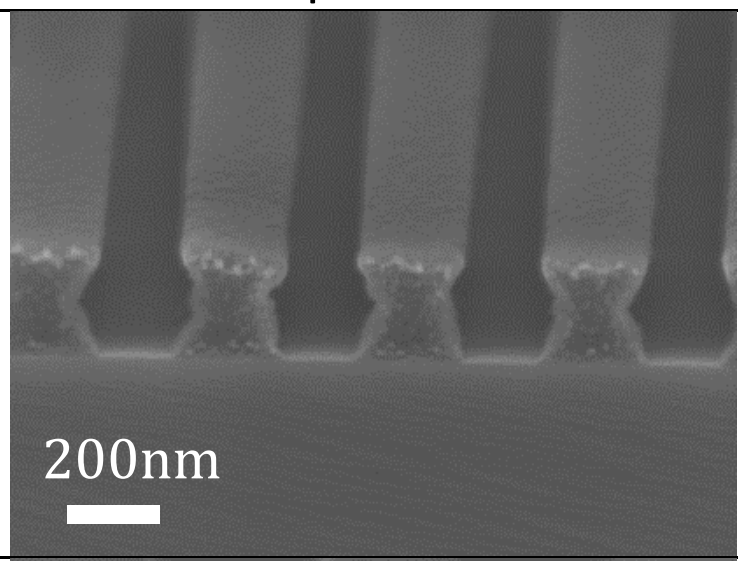	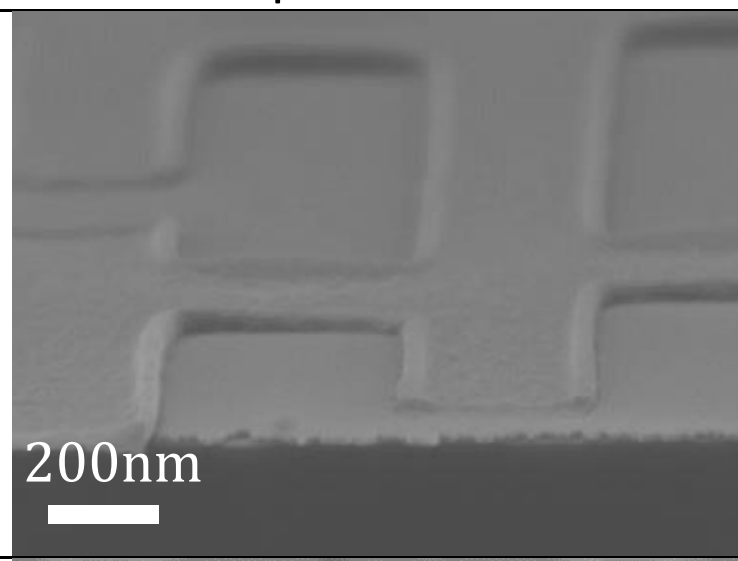
MMA/PMMA bilayer	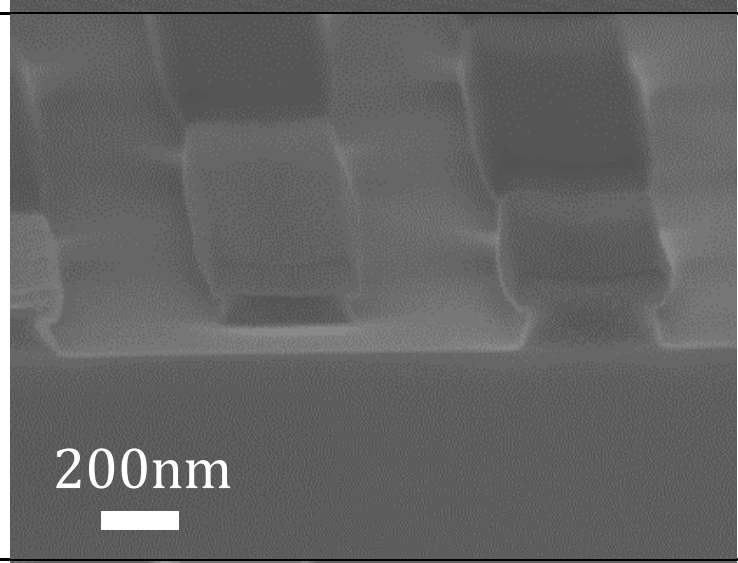	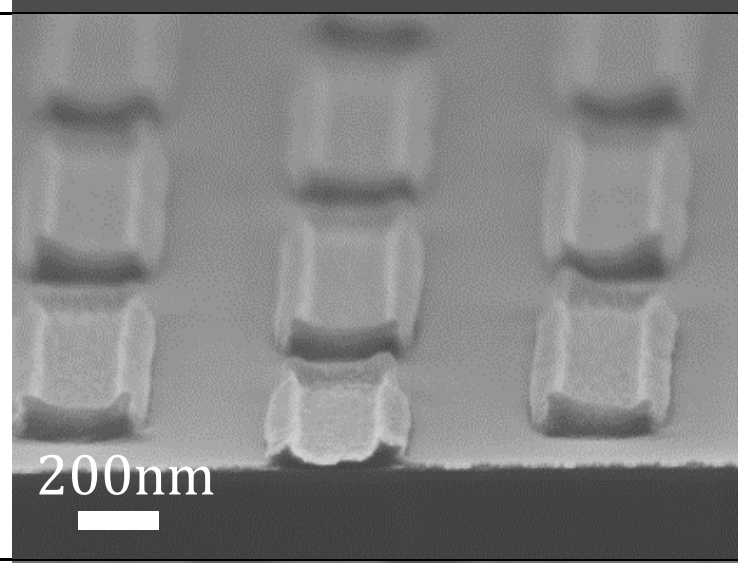
CSAR 62	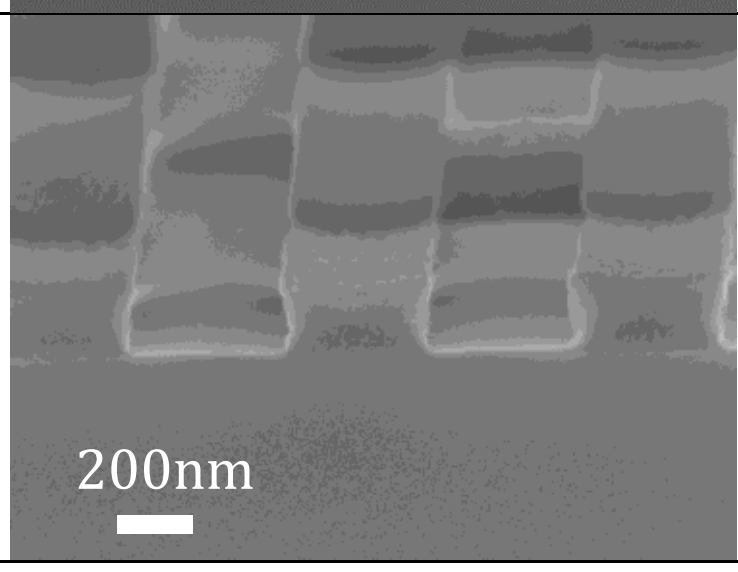	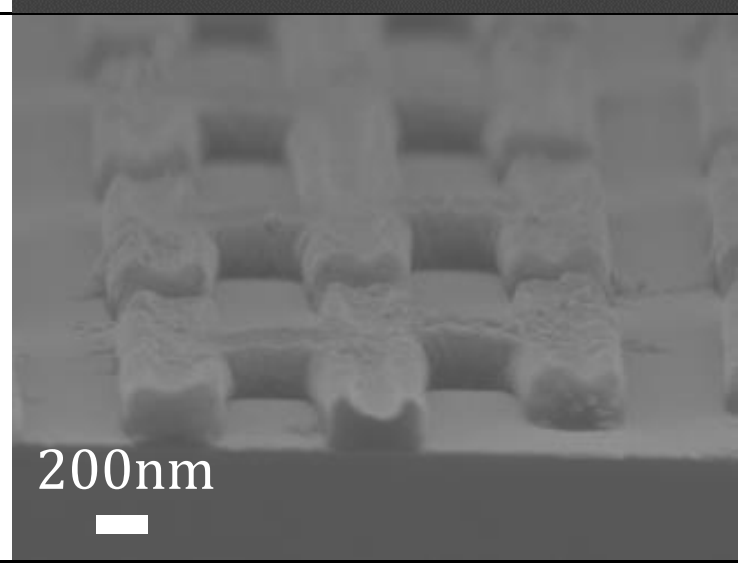

Figure SI 1: *Examples of* different resists *after* Pt deposition. The first column denotes the resist used to pattern the structure in EBL. *C*ross-sectional SEM *images (*second column*)* of the patterned structures *and* after *(third column) a deposition of* 12 nm-*thick Pt film by electron beam assisted evaporation*. Clear deformation is visible in all three samples indicating that the resist is reflowing during *the* Pt deposition. The overall yield and consistency of the lift-off process is low due to the severe deformation of the resist profile.

__**Conventional ways of patterning Pt directly show subpar MacEtch results**__

MacEtch has been performed after patterning with standard lift-off of the catalyst on a limited number of experiments. MacEtch after standard lift-off shows a poor pattern transfer with a lot of defects even in the very early stage of the etching process. In contrast, the interlayer approach has a much larger process window. Figure SI2 presents a direct comparison of standard lift-off and interlayer approach (I-Lo) after the same MacEtch experiment: 1) Pt layer appears to be severely damaged even in the area surrounding the pattern; 2) Pt layer at the bottom of the trenches shows much higher tortuosity in the standard lift-off sample with respect to the I-Lo sample; 3) the number of defects in the etched lines is clearly higher in the in the standard lift-off sample with respect to the I-Lo sample. The pretreatment of the samples was identical. The interlayer sample had a cleaning in Piranha solution as well as prolonged oxygen plasma treatment before Pt deposition. The samples did not undergo any post treatment after Pt deposition, such as thermal dewetting, to disentangle the pure effect of the interface in contact with Pt. The samples were etched together for 15 min at 55°C above HF [50%]. The results show that the standard lift-off sample performs poorly in both open areas outside the pattern (a) and inside the pattern (c) in comparison to the interlayer sample (b,d), demonstrating the improved etching with a clean interface.

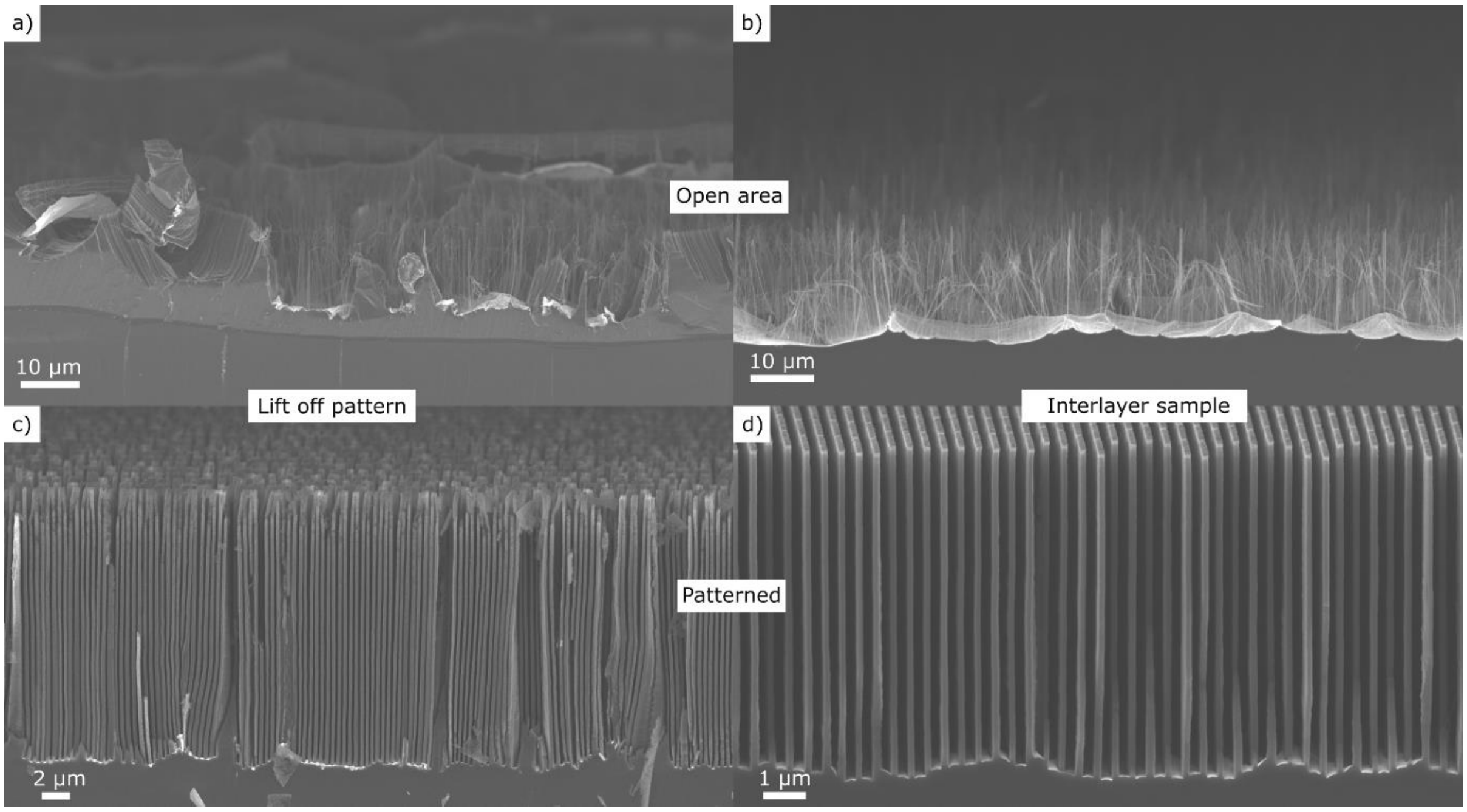

Figure SI 2: Cross-sectional SEM images of a sample prepared with conventional lift-off and one with I-Lo. The I-Lo sample shows improved pattern integrity in both the open and patterned areas.

A second batch of samples were prepared and etched with lift-off using CSAR. The etching was slowed down this time to disentangle the speed. Figure SI 3 shows cross-sectional SEM images of samples etched at 60 °C for 1h over HF[25%]. Even though the etch depth is modest, the structures show clear defects (a) and deviations (b) as well as increased porosity at the top implying a non-clean lift-off.

.

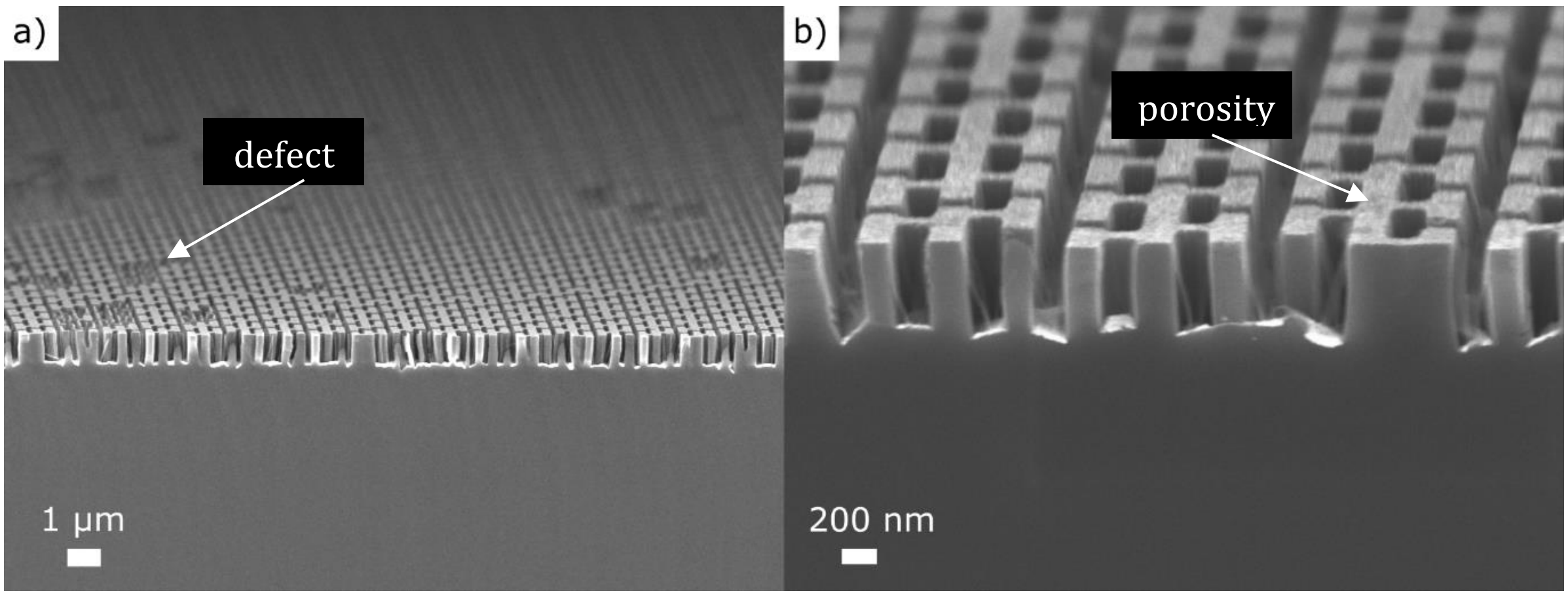

Figure SI 3: a) cross-sectional SEM images of a sample created by standard lift-off with CSAR resist after MacEtch. Clearly visible are defects from poor lift-off on the top as dark areas, where the grating is missing. In addition, there is no straight path of etching even at modest aspect ratio, more visible at b) high magnification.

Ion beam milling with Ar sputtering has been attempted to pattern transfer the pattern into the Pt layer, the pattern presents typical fences indicating a poor quality of pattern transfer, which translates in poor vertical etching, local pinning and high porosity from isolated catalyst motion during a short MacEtch.

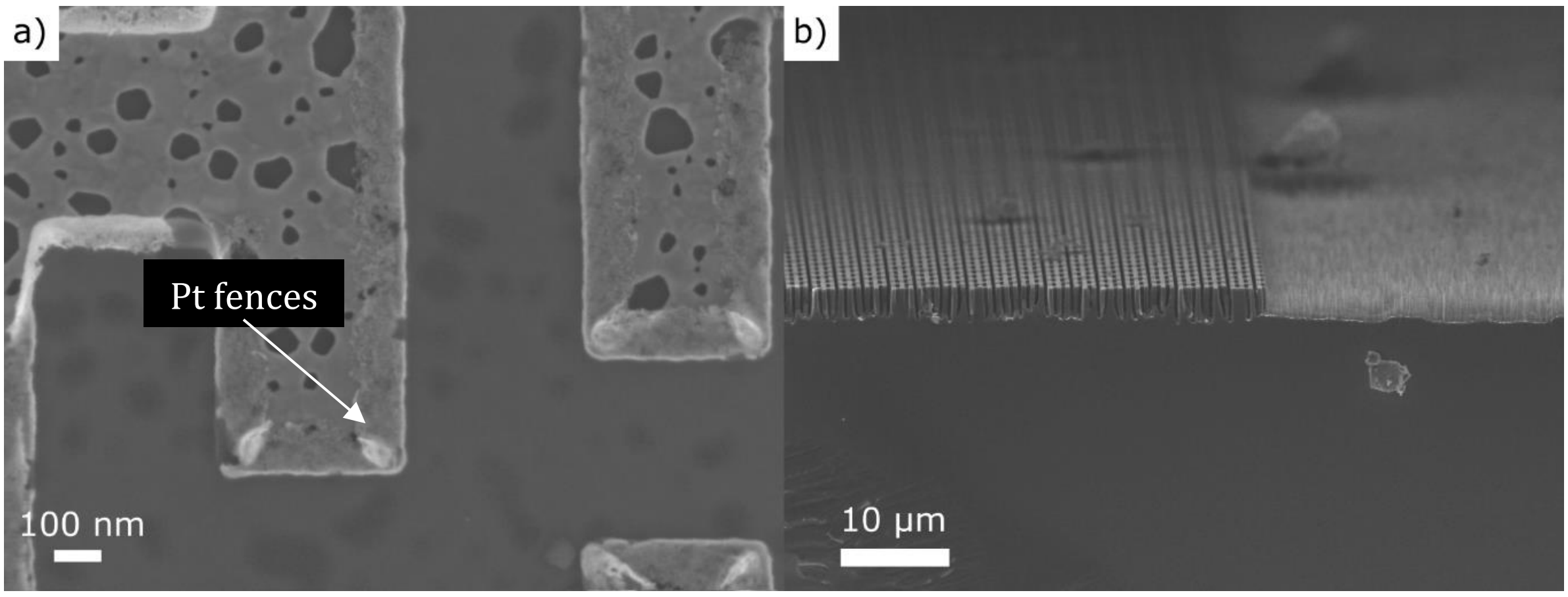

Figure SI 4: sample after Ar ion milling: a) topdown SEM image showing typical fencing on the side from redeposited Pt; b) cross-sectional SEM after MacEtch, indicating no straight etching, local pinning and high porosity from isolated catalyst motion.

Carbon contamination

Even in successful EBL patterning with standard lift-off process using CSAR62 resist, carbon contamination is present after EBL exposure, development and oxygen plasma treatment. We report here few examples supporting this hypothesis. In a patterned sample with standard lift-off, we performed the best oxygen plasma process to clean the surface after the development in compliance with the minimal preservation of the resist profile. After the Pt deposition, we mechanically removed the Pt layer by a strong sonication. This occurs in few random locations of very small area. The SEM images in these locations show an intermediate contrast in the regions previously covered by Pt with respect to Si and Pt, indicating that the oxygen plasma cleaning before the Pt deposition was not enough to return a clean interface. CSAR62 is a positive resist so the Si regions correspond to unexposed resist. The signal in EDX is not sufficient to clearly distinguish C between the regions underneath Pt and the surrounding so we investigated the C contamination using XPS.

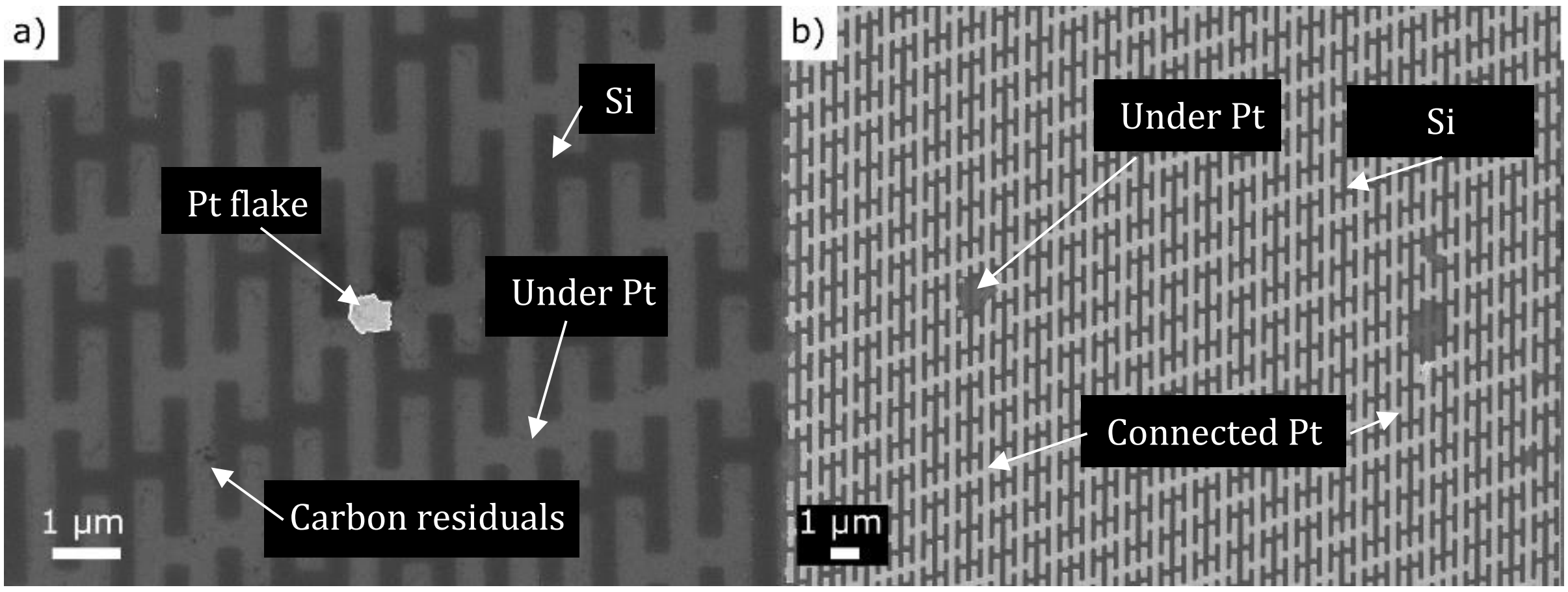

Figure SI 5: a) A top down SEM image of Pt removed by sonication after a standard lift-off. The bright spot in the center is a small flake of Pt remaining. The written h-bar pattern is still clearly visible, even though no Pt is remaining. In addition, black spots can be seen where the Pt is supposed to sit, indicating contamination that is found underneath the catalyst in classical lift-off. This is visible even though the sample underwent an oxygen plasma cleaning. b) top down of one of the more successful patterns by standard lift-off, showing small parts of Pt layer missing: the contrast underneath Pt indicates an uncleaned interface with respect to silicon.

A silicon chip was treated as follows: 1) cleaning in a Piranha solution of H_2SO_4 and H_2O_2 3:1 at 90C for 10 min; 2) spinning of CSAR-62 resist; 3) exposing a full 1 cm x 1 cm area in EBL with a typical dose optimized for structures with 200 nm lines; 4) developing the area to fully remove the exposed resist; 5) typical oxygen plasma descum for 20 s. As a reference, a second Si chip was cleaned together with the EBL sample in the same Piranha bath and the same oxygen descum. Both samples were exposed to air for the same time and went together in the XPS vacuum chamber at the same time. The used XPS is equipped with a monochromator and an analyzer with a focal length of 150 mm, offering high resolution of the peaks. XPS C1s peak is reported in the following Figures for the EBL treated sample (a) and the reference sample (b), the arbitrary units have the same normalization so measuring the area of the two peaks return a 2.56 times higher carbon signal for the exposed sample, showing that carbon is still present, even after oxygen descum. Increasing the descum would lead to a deterioration of the resist profile with a consequence decrease of process yield of standard lift-off. This sets a limit about cleaning level of the interface using a standard lift-off of EBL.

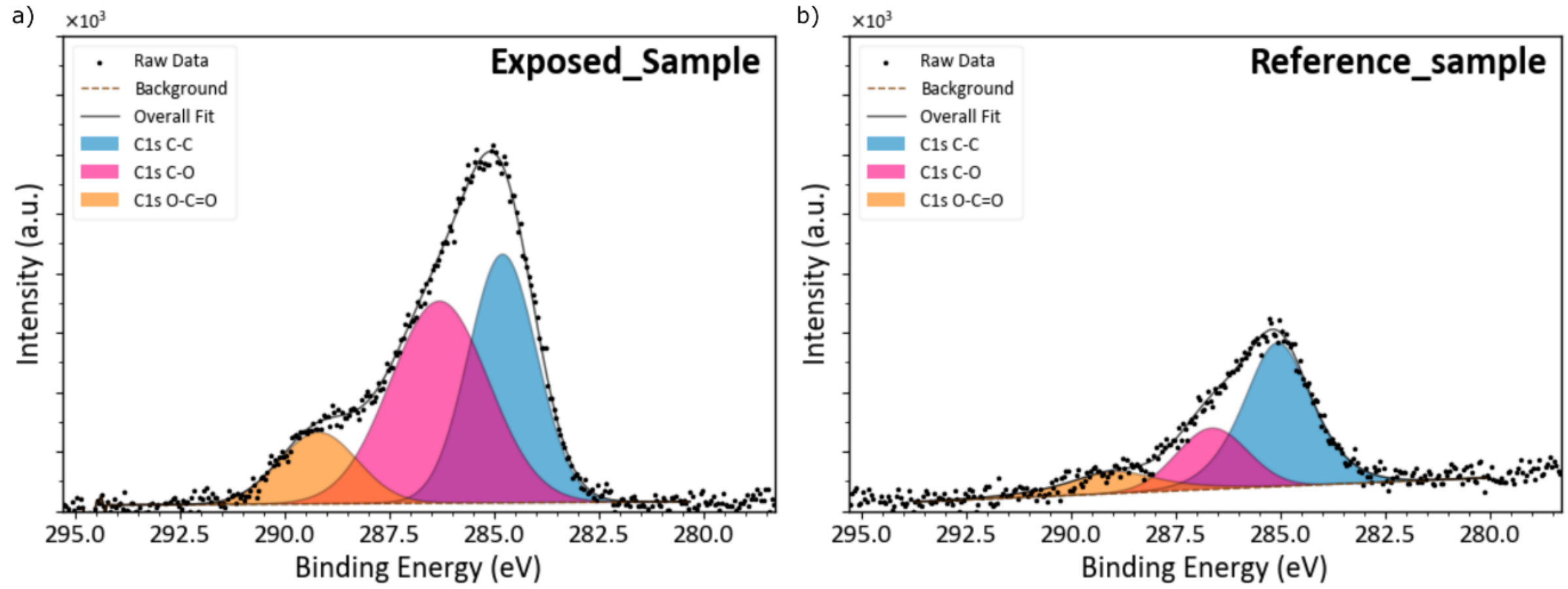

Figure SI 6: XPS analysis a sample that was exposed to EBL, developed and subjected to oxygen plasma cleaning (a) and a reference sample subjected to the same cleaning of the surface (b).

<u>Pinning of the catalyst leads to porosity and removal of the catalyst without subsequent sinking of the catalyst</u>

A central aspect of MacEtch is the mass transport of reactants and byproducts.[1-5] In particular, to remove the Si underneath the thin catalyst film, HF must reach it. The edges of the catalyst pattern provide preferential sites for the HF vapor to easily access the catalyst underneath, constituting faster etching sites that can eventually deform the pattern structure. The relevance of HF mass flow through the catalyst pattern has been demonstrated in previous work by introducing a dewetting process to create a random network of pores in the catalyst layer.[6] Dewetting of the metal catalyst on an oxygen-terminated silicon surface is caused by thermal treatment prior to etching. This causes the catalyst to form pores and cracks at the nanoscale.[7] The extra pores facilitate the penetration of HF through the catalyst, favoring uniform etching. Decreasing the size of the patterned features in the catalyst layer should progressively favor HF diffusion.

Chromium creates an alloy with Pt at elevated temperatures,[8] which conflicts with the temperature required for dewetting. Because Cr is a blocking material for MacEtch, a sample thermally treated at 250 °C with a Cr interlayer does not preserve the pattern transfer during MacEtch, and the etched structures present extended random porosity up to a few 100s nm below the catalyst. If a thermal treatment is required, Cr needs to be removed prior to dewetting.

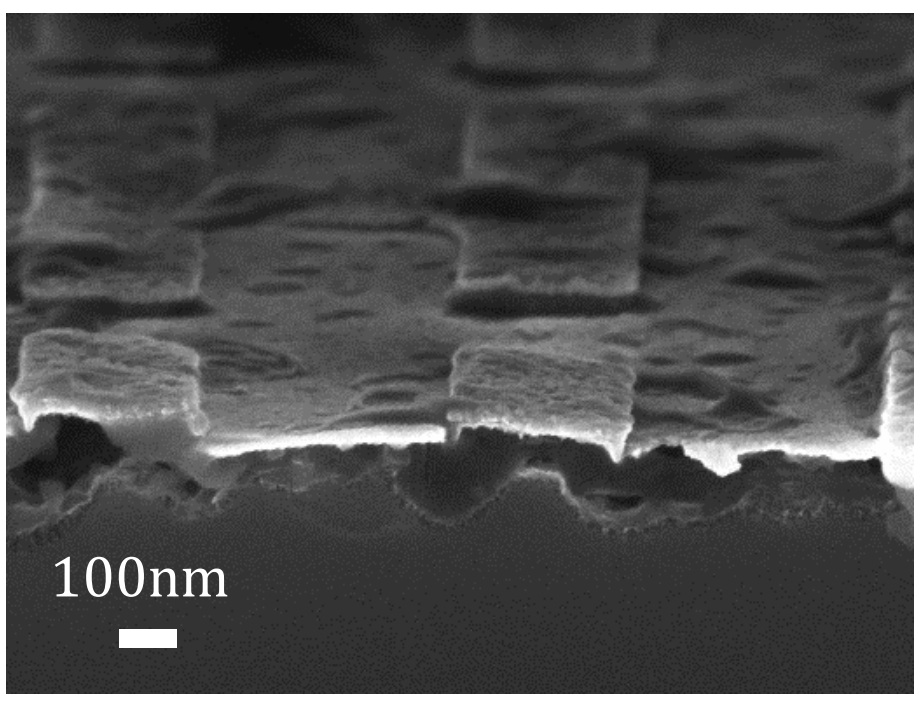

Figure SI 7: A cross-sectional view of a sample dewetted at 450°C without the removal of Cr before dewetting. The result is a large area of pinned catalyst and removal of the Si beneath the catalyst without a clear movement of the catalyst layer inside the silicon substrate typical of MacEtch.

Further option for creating artificial undercut with advanced patterning option

The Cr/Al_2O_3 interlayer can be processed with a liquid etchant or Cl_2/O_2 plasma for lateral thinning the Cr layer. Presented in Figure SI 8 is the undercut after a Cl_2/O_2 plasma demonstrating selective vertical etching of Cr over Si and Al_2O_3

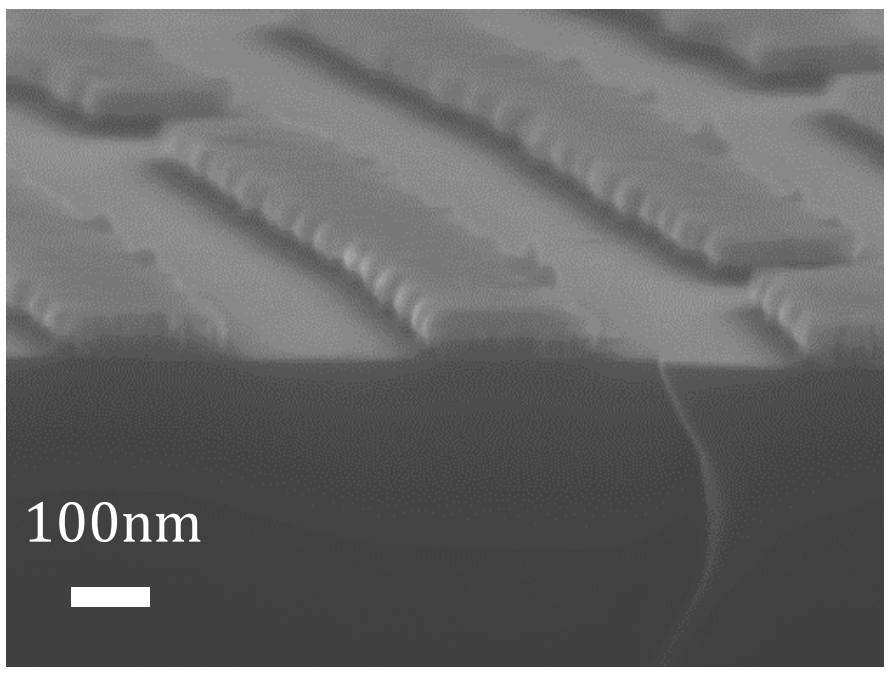

Figure SI 8: SEM in cross section of Cr/ Al_2O_3 interlayer with artificial undercut created by plasma etching. The lower layer (Cr) is laterally etched, using the upper (Al_2O_3) as a hardmask.

Angle and tortuosity measurements

A relevant aspect is the uniformity of the large-scale samples provided in the main text. In the following figure we report an example of angle and tortuosity measurements of the 0.5 cm x 0.4 cm sample presented in Figure 6. The error in the measurement of the etching angle increases with the aspect ratio since the SEM magnification has to scale down to be able to see the top and the bottom of the trenches in the same scan. This support the relevance of quality assessment in the early stage of the etching, with a consequent sacrifice of the sample being cross-sectional SEM a destructive technique. For the tortuosity, we interpolated the etch front by taking a point every 100 nm linearly approximating unseen areas and then dividing the length of the etch front by the direct point to point distance. Etching angle and tortuosity as a function of the different locations in the sample: the expected etching direction is close to 90° with a tortuosity of under 1.1 indicating a good etching profile, apart from the edge.

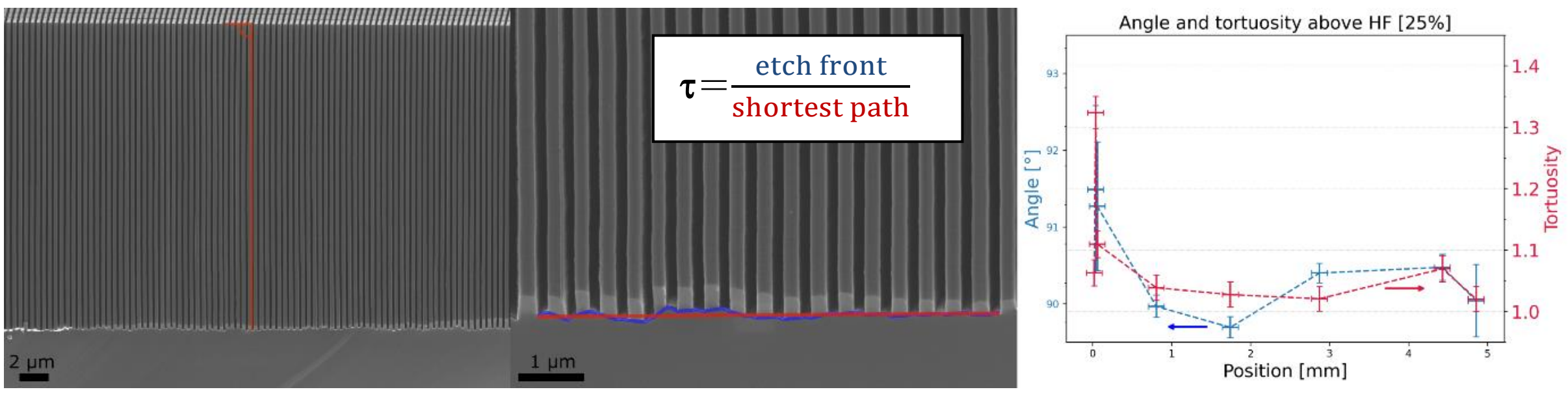

Figure SI 9: Example measurement of etching angle a) and tortuosity b) in cross-sectional SEM images. Angle and tortuosity measurements of the sample presented in the main text in Figure 6 as a function of the position in the pattern c).

Second run of a 1 cm x 1 cm sample etched with MacEtch shows worse pattern transfer, but no complete breaks

The sample of the second run in Figure 6 showed less pattern integrity with multiple isolated breaks and etchings defects visible in the cross-sectional SEM. However, it still possible to measure the etching depth for an overall profile measurement over the large area of the sample.

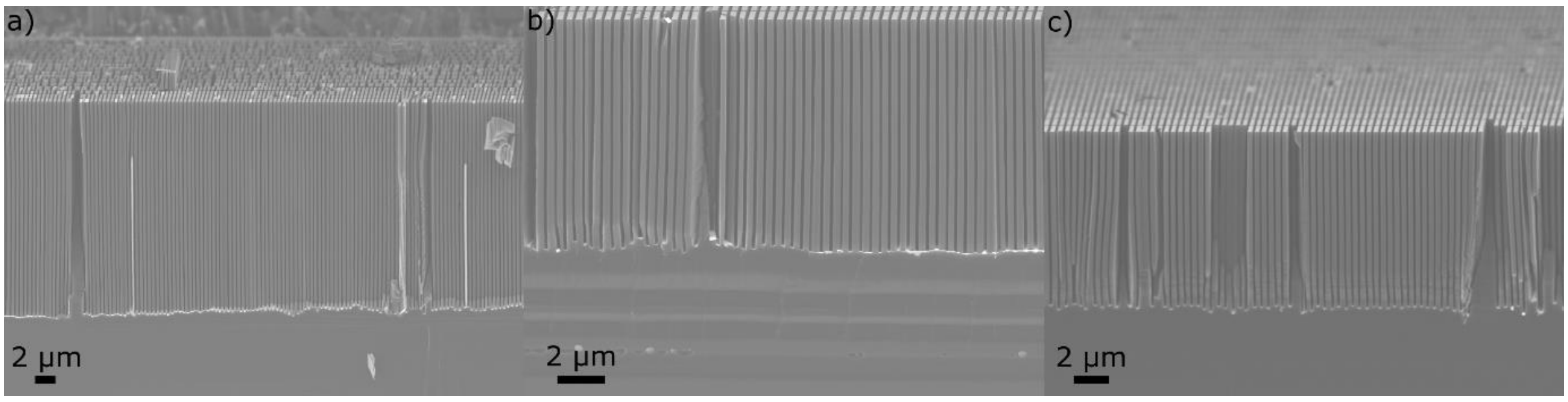

Figure SI 10: Cross-sectional SEM images of the second 1 cm x 1 cm sample etched in Figure 6. Local defects in the patterning decrease the quality compared to the first etched area reported in Figure 6.

Since a third attempt eight months later showed worse results and breaking, we suspect an aging effect of the samples. This could be due to defects stemming from Cr alloying, worse Cr removal over time, or progressive environmental contamination diffusing beneath the catalyst, manifesting as degradation of the catalyst activity with age. A detailed analysis of the aging effect is out of the scope of this work since it would require a well-defined and controlled environment for the sample storage or a dedicated process for faster aging.

Schematic of the IDONUS tool used

The used tool is a commercially available vapor HF tool from IDONUS. The sample is clamped to the heated lid of the tool. The oxygen is available from the ambient air and the HF is evaporated from a liquid reservoir that is flown into the chamber after closing it.

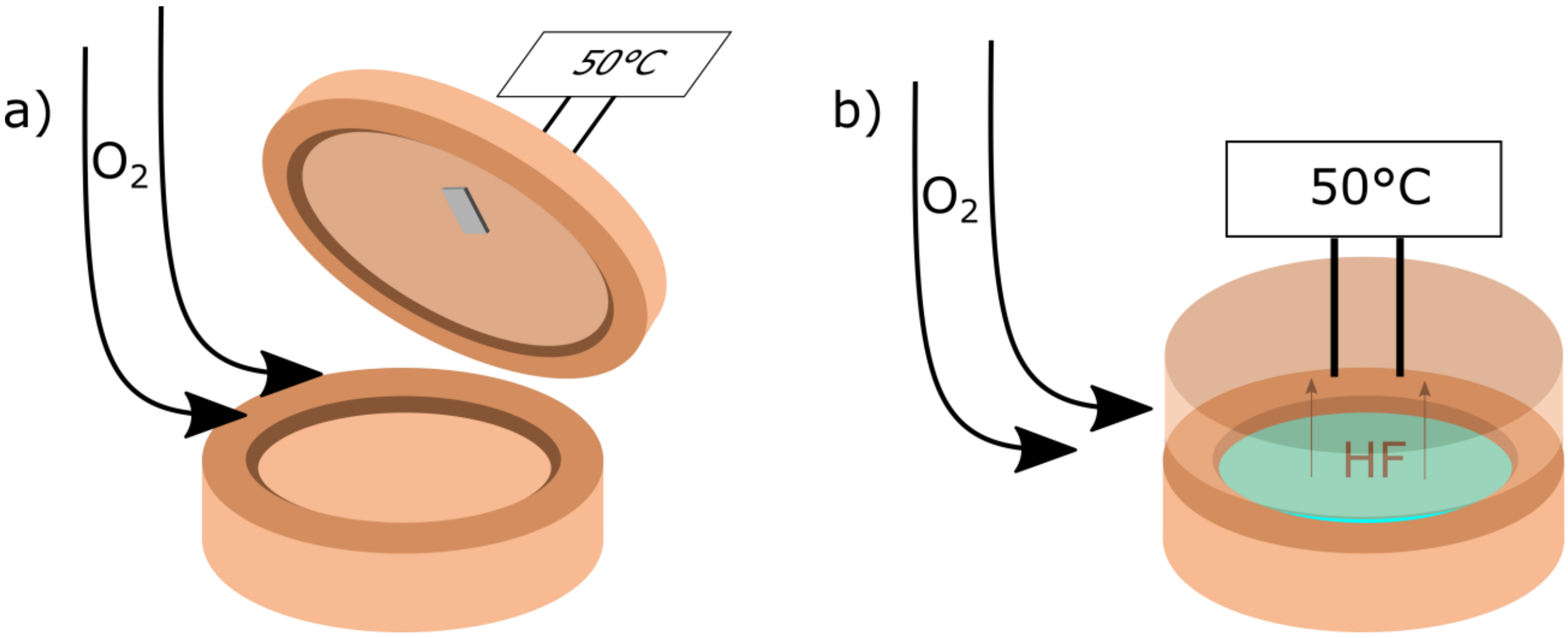

Figure SI 11: A sketch of the used vapor HF tool from IDONUS. The sample is placed on the top part of the tool a), which can be heated. The tool was then closed, and HF was flown in (b). The sample is held at a distance of 1.3 cm from the liquid allowing HF to evaporate in the chamber and oxygen to flow into from the side.

References

1 O. J. Hildreth et al., Vapor Phase Metal-Assisted Chemical Etching of Silicon, Advanced Functional Materials 24 (2014) 3827-3833

2 U. Han-Don et al., Versatile control of metal-assisted chemical etching for vertical silicon microwire arrays and their photovoltaic applications, Scientific Reports 5 (2015) 11277

3 T. K. Kim et al., Bulk Micromachining of Si by Annealing-Driven Magnetically Guided Metal-Assisted Chemical Etching, ACS Applied Electronic Materials 2 (2020) 260-267

4 Z. Huang et al., Metal-Assisted Chemical Etching of Silicon: A Review, Advanced Materials 23 (2011) 285-308

5 N. Geyer et al., Model for the Mass Transport during Metal-Assisted Chemical Etching with Contiguous Metal Films As Catalysts, The Journal of Physical Chemistry C 116 (2012) 13446-13451

6 L. Romano et al., High-Aspect-Ratio Grating Microfabrication by Platinum-Assisted Chemical Etching and Gold Electroplating, Advanced Engineering Materials 22 (2020) 2000258

7 C. V. Thompson. Solid-State Dewetting of Thin Films, Annual Review of Materials Research 42 (2012) 399-434

8 M. Seifert et al., Capability Study of Ti, Cr, W, Ta and Pt as Seed Layers for Electrodeposited Platinum Films on γ-Al2O3 for High Temperature and Harsh Environment Applications, Materials 10 (2017) 54
